# BiologicalNetworks 2.0 - an integrative view of genome biology data

**DOI:** 10.1186/1471-2105-11-610

**Published:** 2010-12-29

**Authors:** Sergey Kozhenkov, Yulia Dubinina, Mayya Sedova, Amarnath Gupta, Julia Ponomarenko, Michael Baitaluk

**Affiliations:** 1 San Diego Supercomputer Center, University of California, San Diego, 9500 Gilman Drive, La Jolla, CA, 92093, USA; 2 Skaggs School of Pharmacy and Pharmaceutical Sciences, University of California, San Diego, 9500 Gilman Drive, La Jolla, CA, 92093, USA

## Abstract

**Background:**

A significant problem in the study of mechanisms of an organism's development is the elucidation of interrelated factors which are making an impact on the different levels of the organism, such as genes, biological molecules, cells, and cell systems. Numerous sources of heterogeneous data which exist for these subsystems are still not integrated sufficiently enough to give researchers a straightforward opportunity to analyze them together in the same frame of study. Systematic application of data integration methods is also hampered by a multitude of such factors as the orthogonal nature of the integrated data and naming problems.

**Results:**

Here we report on a new version of BiologicalNetworks, a research environment for the integral visualization and analysis of heterogeneous biological data. BiologicalNetworks can be queried for properties of thousands of different types of biological entities (genes/proteins, promoters, COGs, pathways, binding sites, and other) and their relations (interactions, co-expression, co-citations, and other). The system includes the build-pathways infrastructure for molecular interactions/relations and module discovery in high-throughput experiments. Also implemented in BiologicalNetworks are the Integrated Genome Viewer and Comparative Genomics Browser applications, which allow for the search and analysis of gene regulatory regions and their conservation in multiple species in conjunction with molecular pathways/networks, experimental data and functional annotations.

**Conclusions:**

The new release of BiologicalNetworks together with its back-end database introduces extensive functionality for a more efficient integrated multi-level analysis of microarray, sequence, regulatory, and other data. BiologicalNetworks is freely available at http://www.biologicalnetworks.org.

## Background

As substantial amounts of data concerning expression, interactions/pathways, sequences, and other types of information for a variety of tissues, developmental stages, stimuli and organisms are generated, it becomes difficult for researchers with no background in bioinformatics and statistics to extract the information they seek. Successful data integration is hampered by the orthogonal nature of the integrated data and by the multitude of controversies and name/ID conflicts in public databases. Examples of conflicts that cannot be automatically resolved include the situations when genes with the same name point to different chromosome locations or a gene/protein in different modification states has different names; for example, p53, p53(361-393), p53(modified:Thr:212), or pCMX-mutant-p53. Among the name/ID conflicts that can be resolved is, for example, the conflict between different genes/proteins having the same synonym or the conflict between two databases naming the same gene differently - these and similar name/ID conflicts can be automatically resolved if there are other databases that recognize the conflicting names. To analyze and visually integrate publicly available data on the systems level, several web-based tools have been developed: Genomatix [1,2], GeneGO [3], STRING [4], Cytoscape [5], VisANT [6], Ingenuity [7], Pathway Studio [8], PipelinePilot [9], and BiologicalNetworks [10]. Workflow systems, like Taverna [11], GenePattern [12] and Galaxy [13], have been designed for the automatic application of the computational methods and data provenance management rather than visual integration, representation, querying and analysis of the data which are addressed in BiologicalNetworks. Each of the mentioned tools has a distinct set of features, which are highlighted in Table 1, facilitating functional analysis of networks/pathways as well as comparative gene sequence analyses, including cis-element prediction, expression profiling and co-expression analysis.

**Table 1:** Web-accessible tools for microarray pathway and DNA sequence regulation analysis.

Features/Tools		GG	PS	ST	IN	PA	CS	GE	VS	GX	TV	PP	BN
**Pathway/Networks**	Curated Pathways Content	+	+	-	+	-	-	-	-	-	-	+/-	+/-
	
	Biological Themes/Functional Enrichment	+	-	-	+	-	+	-	+	-	-	-	+
	
	Build Pathways/Networks inference	+	+	+	-	-	+	+	+	-	-	-	+

**Microarray**	Multi-Experiment Support	-	+	-	+	+	-	-	-	+	+	+	+
	
	Search of Public Expression Compendiums	-	-	-	-	+	-	-	-	+/-	+/-	+	+
	
	Microarray-Pathway-Sequence Integration	-	-	-	-	+	+/-	-	-	+/-	+/-	-	+

**DNA Sequences**	General	+/-	-	+	-	+	-	+	-	+	+	+	+
	
	Gene Regulation	+	-	+	-	+	-	+	-	+/-	+/-	+	+
	
	Regulatory regions	-	-	-	-	+	-	+	-	+/-	+/-	-	+
	
	Sequence search	-	-	-	-	-	-	+	-	+	+	+	+/-
	
	Sequence Annotation	-	-	-	-	-	-	+	-	-	-	-	+/-

**Comparative Genomics**	Homology Search	-	-	+	-	+	-	+	-	+	+	-	+
	
	Search for homologous TFBS^#^	-	-	-	-	+	-	+	-	-	-	-	+

**3D Structure/Drug design**	Visualization	-	-	+	-	-	-	-	-	-	-	+	+
	
	Ligand search	+	+	+	+	-	-	-	+	-	-	+	+

**Back-end Database**	General	+	+	+	+	+	-	+	+	+/-	+/-	+	+
	
	Integration of user's data	+	?	-	?	?	-	-	-	-	-	+	+
	
	Scalability	-	?	+	?	?	-	?	-	?	?	?	+
	
	OBO ontologies integration	-	-	-	-	-	-	-	-	-	-	-	+

**General**	Project Workspace, Data Sharing	+	+	-	+	+	-	+	+/-	+	+	+	+
	
	API/Plugins	+	+	-	+	+	+	-	+	+	+	+	+/-
	
	Free for Academic Use	-	-	+	-	-	+	-	+	+	+	-	+

IN, Ingenuity; GG, GeneGO suite; PS, Ariadne Genomics Pathway Studio; GE, Genomatix suite; ST, STRING; CS, Cytoscape; VS, VisANT; PA, Partek; PP, PipelinePilot; GX, Galaxy; TV, Taverna; BN, BiologicalNetworks.

+, a feature is present; -, not present; +/-, present but not fully; ?, unknown/cannot be determined;

#, TFBS, transcription factor binding site

In this work, the application BiologicalNetworks 2.0 for integration of functional genomics data with biological networks is presented. In comparison with other tools (Table 1; only features that are present in BiologicalNetworks are shown), BiologicalNetworks integrates much more different types of data and provides broader analytical capabilities. The developed system minimizes the need for prior knowledge of existing nomenclatures and data formats representing microarrays, networks/pathways, sequences, and other types of data. BiologicalNetworks allows integral visualization and analyses of over 100,000 features from multiple different data types that are integrated in IntegromeDB [14,15] and provide information concerning pathways, molecular interactions, gene function, expression data, sequences, transcription factor binding sites, promoters and other gene regulatory regions, orthology, mutations and disease relations for thousands of eukaryotic, prokaryotic and viral organisms. Querying capabilities implemented in BiologicalNetworks allows accessing all integrated data simultaneously, from specified databases only, or in conjunction with the user's data. BiologicalNetworks provides an interactive and user-friendly interface with a strong emphasis on graphical data representation. This paper aims to describe BiologicalNetworks 2.0 and its application to navigating through the sea of integrated data and extracting biological knowledge.

## Implementation

BiologicalNetworks 2.0 is built on the NetBeans platform from SUN Microsystems [16]. The system has a modular architecture and an intuitive and customizable user interface and has been tested for robustness to system failure and big data loads. A typical user of BiologicalNetworks loads large (GBs) datasets from files and analyzes these data in conjunction with publicly available data integrated in our database. Data uploads to the program are limited only by the user's computer capabilities. Now, by default, a 32-bit Java can upload (from local files or from the database) ~2GB of data, and 64-bit Java has practically no limits and can go up to 64 GB (and more) in modern computers.

### BiologicalNetworks integrated database

The backend database of BiologicalNetworks, called IntegromeDB [14], is a semantic graph base 'deep-web' data integration system, or data warehouse, that automatically captures, integrates, and manages publicly available data concerning transcriptional regulation, along with other relevant biological information. IntegromeDB integrates over 100 experimental and computational data sources providing genomics, transcriptomics, genetics, functional and interaction data in eukaryotes and prokaryotes. The example databases integrated in IntegromeDB include NCBI nucleotide and protein databases [17], metabolic and signaling pathway databases, such as KEGG [18], interaction networks database, such as STRING [4], and databases of transcription factors and gene regulatory regions, such as TRANSFAC [19] (the full list of integrated databases is provided at http://www.biologicalnetworks.org/Database/tut5.php). IntegromeDB data is stored in a Postgres database under the MetaGraph schema and is updated monthly, being synchronized with the latest changes in most current databases. Detailed information on the statistics, integrated databases catalog, and organism list can be found at http://www.integromedb.org.

The procedure of data integration and mapping to the internal database is fully automated and is based on the Semantic Web technologies, such as the Resource Description Framework (RDF) http://www.w3.org/RDF/ and the Web Ontology Language (OWL) http://www.w3.org/TR/owl-ref/. IntegromeDB also enables researchers to integrate their own data into the database as described in the section 'Integration of User's Data.'

The IntegromeDB schema is based on BioNets Ontology, the core of which is Basic Ontology that was manually developed by the authors. Basic Ontology describes classes from different domains, such as, protein, gene, pathway, interaction, disease, cell, tissue, drug, chromosome, COG functional group, gene set (e.g., operon, regulon). Basic Ontology is manually mapped onto 25 OBO ontologies, including Sequence Ontology, GeneOntology, Human Disease, CheBI, BRENDA Tissues, that were selected from the best curated and regularly updated ontologies provided by the OBO consortium http://www.bioontology.org. The mapping among the OBO ontologies, which is provided by OBO, allows for the automatic integration of 98 ontologies in BioNets Ontology. The basic.owl file with Basic Ontology and mappings from it to other ontologies can be downloaded at http://www.integromedb.org/bionetsonto.php.

Due to the generic schema of the database and ontology-driven mapping, during integration, new objects and their properties are automatically added in the database. For example, if the database stored information about the interaction between the two objects, proteins × and Y, new information about this interaction will be integrated as a new property(s), e.g., a property 'p-value from the experiment A', and the 'experiment A' will be added in the database as a new object. If a clear evidence of, or reference to, a class from the BioNets ontology is absent (missed), an automatic procedure to statistically evaluate the content of the integrated table and assign a term from the ontology is applied. The procedure takes each word and word combination in the table, search for them in the BioNets ontology, calculates the statistical significance of the occurrence, and assigns the most significant term to the table.

Upon the integration of a new data source, the database automatically identifies conflicts in names, name synonyms and IDs of all objects in the database (genes, proteins, organisms, etc.) among various databases and identification systems. All names/IDs are weighted and sorted by the number of integrated data sources, supporting the name/ID. When the user searches the database, names/IDs appear in the search result in the descending order by weight; the potential conflicts will appear in the bottom of the list. Thus, if one database, for example, names the specific gene as × and another database, as Y, both names, × and Y, will be equally searchable; while in the search result, the name that is the most common among all integrated databases will appear first. However, no conflict data are removed or become invisible to the user, since the data sources are not weighted or judged. Inconsistencies among the names/IDs of all objects in the database that were found for human, mouse and rat are provided at http://www.integromedb.org. If the user searches the http://integromedb.org page for an object for which inconsistencies were calculated and found, they can be seen on the result search page by clicking the 'red button'.

The IntegromeDB data can be searched at http://www.integromedb.org; however, in comparison with the application BiologicalNetworks, described in this work, the web site provides only general quick search capabilities and no extensive data analysis, dynamic integration, and visualization capabilities.

### Integrative View of data in BiologicalNetworks

**Figure 1:**
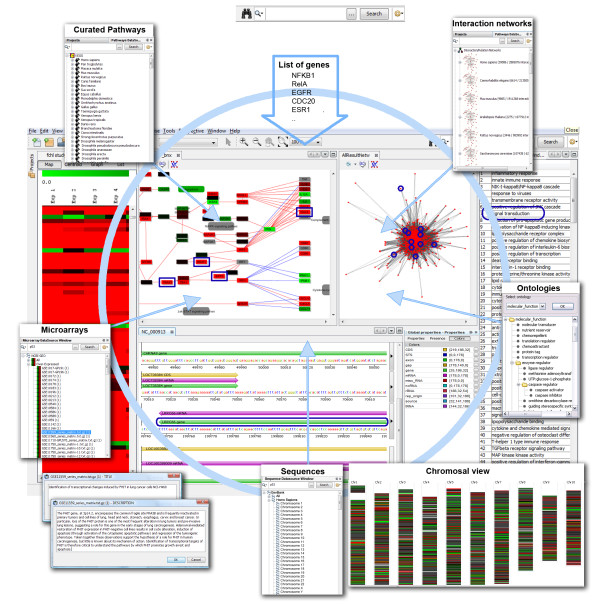
**Integrative view of data in BiologicalNetworks**.

The typical user of BiologicalNetworks starts with loading the file or searching the integrated database for a list of genes (e.g., tab-delimited text-file), networks (.sif-file), curated pathways (.SBML), microarrays, proteomics data (tab-delimited file), or sequence data (.gbk, FASTA, or .gbs-file). BiologicalNetworks provides an Integrative View of the found data (Figure 1), opening the modules of the system (Network, Sequence, Microarray, Ontology, and other modules) in separate windows. All opened modules are synchronized and interconnected by object/gene/protein IDs, so that changing the state of the object/gene/protein in one module of the system automatically changes the states of the respective objects/genes/proteins in all opened modules. This makes it possible, for example, to map color-coded expression data or ontology annotations onto any collection of genes/proteins as well as pathways and interaction/relation networks or chromosomal sequence view that are currently opened in the project. The modules are described in detail in the subsequent sections.

### Microarray data search and analysis

Most current web-based tools are focused on retrieving expression and/or co-expression patterns for individual genes from particular microarray experiment. Multi-experiment/multi-gene co-expression analysis is a labor-intensive and computationally challenging task, involving collecting suitable datasets, data downloads, preprocessing, normalization, and gene annotation management, the integration of different datasets, merging cross-platform data, and handling ambiguous mappings between genes and probe sets. Microarray databases Gene Expression Omnibus (GEO) [20] and ArrayExpress [21] provide tools for finding and analyzing most relevant datasets, but neither currently provides a comprehensive gene co-expression search over many datasets simultaneously. We have refined and extended the process of multi-experiment/multi-gene co-expression analysis in the Microarray Analysis window. BiologicalNetworks database integrates tens of thousands microarray experiments from GEO, ArrayExpress and other public data sources (see the Nucleic Acids Research list of databases on microarray data and other gene expression data [29]). Data from every experiment are normalized, so every expression vector is subtracted from the mean and divided by the standard deviation of the experimental expression values. Since different experiments have different numbers of time points and conditions, the Pearson correlation calculation is FDR-corrected so that the p-Values calculated for PCC take the length of the expression vectors into account.

The user can upload the microarray data files and analyze them in conjunction with the integrated compendium of publicly available microarray data using the Microarray Analysis window. When searching in the Microarray Analysis window, the user can select the 'Default', 'Co-expression pairs' or 'Co-expression Triples' search modes. In the first case the search will return all available microarray experiments in which genes from the input gene list are over- or under-expressed. In the second case, it will return experiments and gene pairs where genes are co-expressed; and in the third case, it will return gene triples and experiments where these gene triples are co-expressed. This last option is especially important for discovering gene regulatory modules (*e.g. *a set of transcription factors regulating a set of co-expressed genes), since several studies exploit the fact that co-expressed and/or functionally related genes may be transcriptionally coordinated [22-24] (see the Demonstration study below). The results are presented in highly interactive graphical format with strong emphasis on further data mining. In the resulting tree the user can pick the datasets manually and visualize it in the main window with the description of the experiment, annotations and metadata. Datasets in the query results can be ordered by highest co-expression to input genes. In addition, to search functionalities the platform provides the user with clustering and functional enrichment analysis tools.

### Integrated visualization of Biological 'Themes' and gene list enrichment analysis

**Figure 2:**
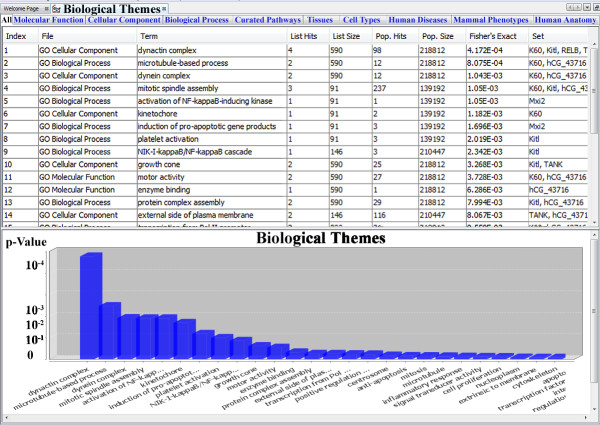
**Biological Themes**. Biological Themes (GO terms, diseases, cell types, tissues, curated pathways, phenotypes and human anatomy) representation in BiologicalNetworks.

The implementation of the Biological 'Themes' within the BiologicalNetworks framework provides a set of tools for giving the researcher a biological interpretation of gene clusters based on the indices provided in the input data set and information linking those indices to biological 'themes' (*e.g. *GO, cell types, diseases, *etc*.). Our gene list functional enrichment analysis currently uses as many as 8 annotation categories including 3 categories of GO terms, curated (e.g. KEGG) pathways, diseases, cell types, tissue gene expression, phenotypes and human anatomy (Figure 2). Hypergeometric distribution with Bonferroni correction is used as the standard method for determining statistical significance [27]. The result of the analysis is a group of biological themes that are represented as gene clusters by themes. Statistical reports show the probability that the prevalence of a particular theme within the cluster is due to chance alone given the prevalence of that theme in the population of genes under study.

Although ontologies are stored as DAGs (directed acyclic graph) in our internal database, we visualize them as tables ('Themes' Tables) for the sake of usability and easier navigation (Figure 2). Since BiologicalNetworks provides Integrative View of the visualized data, all windows of the system and data inside them are synchronized and interconnected by IDs. In the 'Themes' Tables, this is exemplified by the simultaneous highlighting of all gene/protein instances in all opened pathways, networks, experiments, sequences *etc*. representing the same 'Theme' whenever one of them is chosen. Terms under different categories are highlighted using different colors. All clusters of genes/proteins discovered for enriched biological terms are organized by 'themes' under the subfolder Analysis in the Project Panel on the left. Clicking on the expansion symbol or double-clicking over Project Panel tree nodes will expand or collapse it. Other information, such as the number of genes directly annotated under the term, connections microarray experiments, network, sequence data if any *etc*. are also provided.

### Sequence Annotation and Sequence Analysis Browser

**Figure 3:**
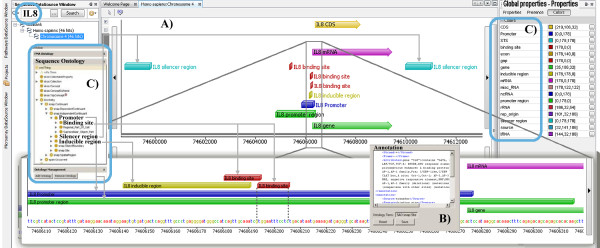
**Genomic Sequence Analysis Browser and Annotations**. **(A) **Using the Sequence Search (at the left) box, the user finds genes in Sequence Browser. In addition to gene related information, all regulatory regions integrated in the database are represented in Sequence Analysis Browser. (**B) **In addition to annotations integrated from public databases, BiologicalNetworks allows users to annotate genomic sequences. Annotation Tab and two ontologies used for annotation are represented. (**C) **Sequence intervals are connected to BioNets Ontology (in this case to Sequence Ontology) nodes through the internal Objects and Attribute values tables that list objects and attributes of different types integrated in the BiologicalNetworks database.

Sequence data together with annotation data, including binding sites, promoters, and other regulatory regions, that have been integrated in the BiologicalNetworks database represent the collection of interval trees; a single interval tree is created per chromosome instead of per annotated DNA sequence regions. Nodes of the interval (RI)-trees, or sequence intervals. are connected to the BioNets Ontology http://www.integromedb.org/bionetsonto.php nodes (Figure 3C) through the internal Objects and Attribute values tables that list a huge amount of objects and attributes of different types integrated in the BiologicalNetworks system. BioNets Ontology is implemented as a directed labeled meta-graph data structure that serves as a general-purpose 'labeled join index'. The general-purpose OWL schema of the BioNets ontology integrates Sequence Ontology, GeneOntology, BioPAX, Disease Ontology, Chemical Ontology, the Functional Genomics Ontology (FUGO) and the Phenotype and Trait Ontology (PATO) and other ontologies provided by OBO consortium at http://www.bioontology.org. It is implemented so that any individual ontology describing another type of biological knowledge (for example epidemiology or pharmacology *e.g. *PharmGKB) can be introduced and modified with minimal impact on the rest of the system. This is implemented through 'ontology mapping' [30]: for every new adapted ontology (for ex. SequenceOntology) that maps a class (for example class *Gene*, 'SO:12345') a new class that maintains mapping to source ontology is generated (i.e. class 'mappingSO:012345') which is connected to a *Gene *in our BioNets ontology through 'same_as' relation. This is done to not modify BioNets ontology classes every time new ontology is ingested, thus while 'unifying' different biological data types to keep specificity of every member schema of our integrated database. Examples of operations on RI-trees that will apply on all substructures (e.g. sequence intervals), called *SUB_X*, are represented below:

*ifOverlap *function: SUB-X * SUB_X - > {0, 1}, returns *true *if the two interval substructures overlap.

*Next *function: *SUB_X *->*SUB_X *is applicable on data types for which there is a strict ordering on the domain; it returns the sub-structure encountered next in the ordering input substructure. The semantics of 'next' depends upon the data types (sequence, anatomical/geographical region, *etc*.).

*Intersect *function: SUB_X * SUB_X ->*SUB_X*, returns the intersection of two *SUB-X*. This operation is valid for convex data types such as sequences and rectangles.

These operators are extensively used in Sequence Analysis Browser for *Navigation *(scroll upstream/downstream, get_next gene/operon/chromosome or next gene_regulatory region) (Figure 3) and *Annotation *of multiply overlapping gene regulatory regions (binding sites, composite regulatory elements, TATA-box, *etc*.) (Figure 3B). Figure 3 represents different types (binding site, TATA-box, operon, *etc*.) of segment elements and different annotations (properties) integrated from many data sources for one gene or gene upstream region. Genomic Sequences are integrated with the meta-graph schema of Biological-Networks database through an ElementId-ObjectID connection table, where elements are sequence elements, for example, a core promoter, TATA box, or binding site, that are attributed to a particular gene by means of known localization in the gene, according to the GenBank global position. Internal enumerations in the integrated databases-TRANSFAC, for example, provides localization of regulatory regions in respect to the transcription start-are recalculated accordingly. The connection table assigns sequence elements to meta-graph objects, so that sequence elements, represented as a RI-tree structure, become graph objects within the meta-graph database. All heterogeneous data, for example gene properties in the Property Panel (Figure 3 top right), integrated in the meta-graph database thus appear to be mapped on genomic intervals and vice versa. In the result, DNA sequences, molecular interaction graphs, 3D protein structures, images of expression, and other types of data integrated in the BiologicalNetworks become connected and annotated within the same context. The sequence part of BiologicalNetworks integrated database is updated monthly from the primary public servers: GenBank, Ensembl and the UCSC Genome Browser database.

In the Sequence Analysis Browser, the user can upload (from the local files or retrieve from the database) large (GBs) volumes of sequence data and analyze them together with the integrated data on gene sequences, annotations, orthologs and cross-references to the major biological databases displayed in the Sequence Annotation Browser (Figure 3). The browser window allows any sequence region within specific gene loci or whole genome to be displayed (Figure 1), using the set of navigation tools that are functionally similar to the tools in UCSC Genome Browser [28]. The rich collection of sequence features, such as exons, introns, the transcription start site, repetitive elements, conserved sequence regions and transcription factor binding sites, are color-coded in the browser window. The whole genome Chromosome Viewer (Figure 1, bottom) is a chromosomal representation of the entire genome of a sample. This view, integrated with experimental data (e.g. microarray), provides easy identification of large-scale abnormalities and the overall aneuploidy of a sample. The display consists of a number of bars (Figure 1), each representing a chromosome. Each bar is composed of a series of colored linear segments, each representing a probe. The user can click on any clone in the chromosomal viewer to see its clone name and chromosome, and all properties (e.g. RefSeq IDs) from integrated database.fho

### Comparative Genomics Browser

**Figure 4:**
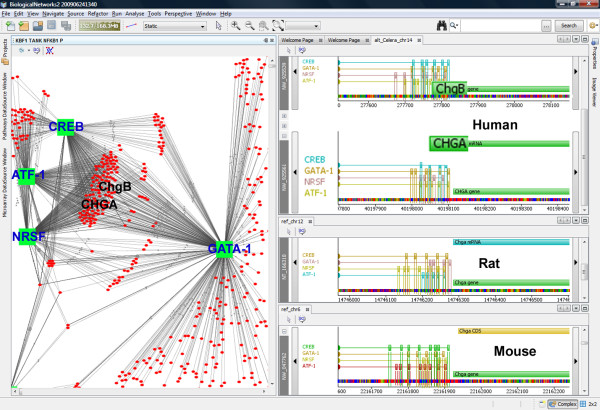
**Screen-shot of Comparative Genomix Browser**. Comparative Genomix Browser shown together with the pathway window that depicts transcription factor-DNA binding network for CREB, NRSF, GATA-1, ATF-1 (green squares) and their targets (red circles). In Comparative Genomix Browser (right window), for selected species, TFBSs collected from public data and predicted using phylogenetic footprinting are shown aligned as provided by phylogenetic footprinting. The user can zoom in/out, scroll the sequences and network regions for respective TFs and genes to see the binding sites, relative chromosomal positions of target genes and respective sequence events.

BiologicalNetworks provides the ability to investigate transcriptional cascades by integrating and visualizing transcription factor gene regulation networks, relevant transcription factor binding sites and target genes with multiple sequence annotations, thus facilitating validation experiments (e.g. primer design applications) (Figure 4). A fundamental problem in building transcription factor (TF)-gene networks based on binding motifs in DNA sequences of putative target genes is the rate of false positive predictions of TF binding sites (TFBSs). To reduce the false positive rate of TFBSs predictions, phylogenetic footprinting methods are used that search only for genomic DNA sequences, which are conserved among species. Phylogenetic footprinting methods still have a high false positive rate because, although a TFBS might be correctly predicted, binding of the respective factor might only occur in certain cells or tissues. The BiologicalNetworks' Comparative Genomics Browser addresses both these problems of TFBS prediction via 1) integrating TFBSs predicted by phylogenetic footprinting methods [25,26]; 2) integrating all available experimental and computational data sources on transcriptional regulation, transcription factor binding sites, together with scores (p-Values) [31] (user can filter interactions by p-value in the Build Pathway Wizard when constructing gene regulatory networks) of binding event for each transcription factor and a gene or a pair of genes; and 3) filtering the final TF-gene interaction networks by tissue- and cell type-specificity in order to correct the probability of binding and thus reduce the false positive rate of TFBSs prediction and narrow the list of potential target genes for further investigation. Described methods can be applied to those genes and species for which data on TFBSs and gene homology are available in the BiologicalNetworks integrated database; if such data are available, gene regulatory regions can be visualized in the browser as it is shown in Figure 4 (right window). The homology information is imported from the COGs database [32]; we extend the COG groups to cover all organisms available in the IntegromeDB database of BiologicalNetworks.

The Comparative Genomics Browser can be explored together with other modules/windows of BiologicalNetworks. For example, Figure 4 shows how the browser can be used in tandem with the network/pathway window on the example of four transcription factors, CREB, NRSF, GATA-1, ATF-1, in three species, human, mouse and rat. Other species, including prokaryotes, can be also studied, subject to data availability. Because if the user intends to explore specific genes/species in these two modules simultaneously, all these types of data, interaction networks/pathways, TFBSs and gene homology, must be available. The browser displays regions surrounding orthologous genes, highlighting orthology relations among them and cases of synteny (co-localized orthologs). The user may change the stringency of evolutionary conservation of TFBSs and apply tissue-specific and cell type-specific filters; in the results, data and their representation visualized in both windows will change synchronically. The Comparative Genomics Browser together with other modules of BiologicalNetworks forms a valuable tool for investigating transcriptional cascades as it is described in the section 'Demonstration Study' below.

### Data Querying

**Figure 5:**
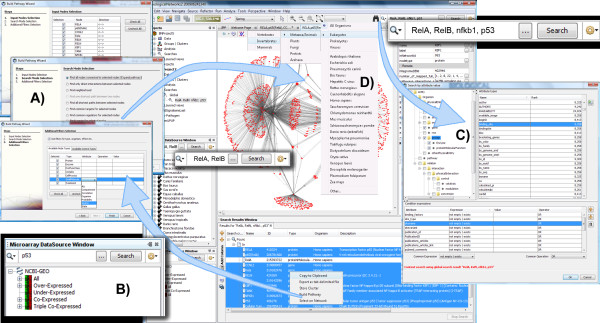
**BiologicalNetworks data querying environment**. **(A) **BuildPathwayWizard (BPW) assists the user in finding regulatory paths and functional links, between selected objects, searches for common targets or regulators for the group of molecules, finds connection to curated pathways (e.g. KEGG). BPW can find functional links between proteins in the lists imported from other programs (e.g. gene expression clusters). (**B) **Specialized Search Boxes are available on the Microarrays, Curated Pathways and Networks panels. (**C) **Search by attributes provides an advanced querying facility for retrieving the data of user's interest by querying Objects and Properties types. User friendly querying interface allows user to make query with any logical combination of conditions both on Node and Property types. (**D) **Organism Filter on 21 mostly used organisms and organism groups.

The BiologicalNetworks interface contains multiple search and build pathways/networks capabilities (Figure 5), allowing the simultaneous querying of and the building of pathways/networks, using microarray or proteomics data, networks, curated pathways, sequences, sequence annotations, gene regulatory regions, and other data. The user can start searching for an entity of interest and then query for relations to that entity. Alternatively, the user can import a list of entities and search for relations among them and other entities in the database.

The seven querying options listed below allow for the specification and search for any logical combination of entities, processes/relations and their properties. The first four options are available from Quick Search Box at the top right corner of the program (Figure 5) and allow searching the entire database. The next three query options define more specific searches; they are accessible by clicking the buttons to the left and right of the search boxes in the correspondent windows (Figure 5A-C). The search results appear in the bottom list panel (Figure 5, bottom).

1. ***Simple Gene/Protein search ***(default search). For example, the search for the three genes/proteins 'p53, egfr, esr1' returns these three genes in all specified species and gene properties. The list of genes/proteins can be uploaded from the file.

2. ***Keyword search***. For example, the keyword search 'p53, egfr, esr1' returns **all **database objects, including experiments, publications, pathways, and **all **properties of all objects that contain either p53, egfr, or esr1.

3. ***Wild card search***. For example, the search 'neurodeg*' returns all objects related to neurodegenerative, neurodegeneration, and all words in the databases beginning with 'neurodeg'.

4. ***Multi-word search***. For example, the search 'obesity diabetes' (no comma separating 'obesity' and 'diabetes'; comma means OR) returns the results for 'obesity AND diabetes' and 'obesity OR diabetes'.

5. ***Build Pathway Wizard: Path Queries***. Build Pathway Wizard contains dozens types of pathway and network searches (in the opened networks/pathways or integrated database) in protein-protein interactions, transcription factor-DNA networks, relational (co-expression, co-citation, etc.) networks and curated pathways (e.g. KEGG) (Figure 5A). The three wizard windows (Figure 5A) allow users to specify:

a) algorithm type for pathway building;

b) select the directionality of relations;

c) types of objects and property values (e.g., specific proteins from specific species or specific database/dataset) to be included in the pathway; and

d) relations (e.g., p-value of the protein-protein interactions for all or selected experiments) to be included in the new pathway.

6. ***Specialized search ***(Figure 5B). These Search Boxes are available on the Microarrays, Curated Pathways and Networks and Sequence panels. They are for quick retrieval the most popular public data sets.

7. ***Search by attributes ***(Figure 5C). The search by attributes allows the user searching the database, as well as user's data files, for specific entities, using many types of data as search conditions. These include, for example, node type, effect (positive, negative, unknown), mechanism (transcription, phosphorylation), tissue type, description, user-defined attributes text, and so forth. (Figure 5C). These types of queries can contain logical operators on the attributes (a detailed description is provided in the online Tutorial).

Search Boxes accept lists of gene names (that can be loaded from files), accession numbers from public databases (SwissProt, UniGene, GenBank, *etc*.) or GEO experiments (Series and Datasets). The system recognizes most of the currently available gene/protein IDs and synonyms for thousands of organisms integrated from over 100 data sources. To perform a search in the microarray, pathways/networks, sequence annotations and PubMed repositories, the user can specify any combination of keywords, including authors names, tissue types, diseases, gene/protein names. The Search Box contains different configuration options and filters and enables limiting searches on specific species, opened network/pathway and sequences. The organism drop-down menus in each search window include 21 model organisms, which are mostly represented in the database, and the following options to narrow down the search and subsequent data analysis: All Organisms, Eukaryotes, Prokaryotes and Viruses. Eukaryotes are subdivided into Plants, Fungi, Protists, Archea, and Metazoa/Animals, which in turn are subdivided into Vertebrate, Invertebrate and Mammals.

### Loading files and Output results in BiologicalNetworks

In BiologicalNetworks, the user can load/open files in the following standard formats:

• networks: SIF (Simple Interaction Format), PSI-MI, Tab-delimited network file, SBML and BiGG model format;

• microarray data: tab-delimited file format, Illumina tab-delimited and Affymetrix file formats;

• sequences: GenBank (GBK, GBS) and FASTA formats.

The results of analysis and visualization in BiologicalNetworks can be saved at any moment as the BioNets XML Project file and then opened at other computers; the user's settings, data files, results of search, built networks, clustering, colorings and all other visualizations will appear exactly how they were at the moment of the file saving. The project can be also saved/exported to the SIF and SVG formats.

### Integration of users' data

BiologicalNetworks allows the user to work with his/her own data, and to integrate them into the system database (IntegromeDB). The integration procedure is different from the procedure of loading/opening data files described above in that the loaded data are available to the user and also to whoever obtains the BioNets XML Project file that includes the user's data. The integration procedure allows the data to be made public; they become integrated in IntegromeDB as any other database and become searchable in the BiologicalNetworks application and at the web-page http://integromedb.org.

The user can integrate the data at http://integromedb.org under "User's Data Integration" menu (Figure S2 in Additional File 1). The data will become public, but unless it is already curated by the data administrators, it will remain 'tagged' as 'uncurated' under the contributor's name. The integration procedure consists of 3 steps (see Figure S2 in Additional File 1): 1) Registration, 2) Data Mapping and 3) Data Integration. Data mapping and integration is done automatically; it follows the same procedure as one described in the section '**BiologicalNetworks integrated database**.' The user can accept or not accept the resulting mapping. To be integrated, the data needs to be in the table format. Any type of data can be integrated, given that they are provided in the tab-delimited format.

### User support and Problem Handling

To address the critical need for user support, we developed Bug and Problem Report Tools. This tool allows the user to report problems or bugs, while working in BiologicalNetworks. During the installation of BiologicalNetworks and the initial run of the program, the user is asked for the agreement for permission to send from his computer any future bug reports. If the user agrees, Bug Reports will be automatically generated and sent to our support server; it will include the environment settings and the last user's steps before the program gave an error. To report the problem, the user needs to use Problem Report Tool that is located in the 'Tools' menu of the program. The *Sun NetBeans *Report and *Bugzilla *mechanisms are employed in the Bug/Problem Report Tools. Our experience shows that most of the user (especially beginners) problems are minor problems that can be quickly fixed by the developers, if the Bug and Problem Report Tools are used.

## Results

The BiologicalNetworks analytical and querying functionality were applied to and tested in a number of different biological systems/projects, both eukaryotic and prokaryotic: host-pathogen interactions, specifically, the influenza and *Streptococcus pneumoniae *-human/mouse/rat interactions [33], 'meta-genomics' CAMERA project [34], yeast meiosis [35], whole-genome metabolic reconstruction in Humans and E. coli [36], parasite studies in *Giardia lamblia *[37], and microbial metabolism in *Thermatoga maritime *[38]. All described projects are accessible from BiologicalNetworks main page under "Driving Projects" panel or upon launching the BiologicalNetworks application, on the Welcome Screen. These studies can be replicated by running the respective projects.

**Figure 6:**
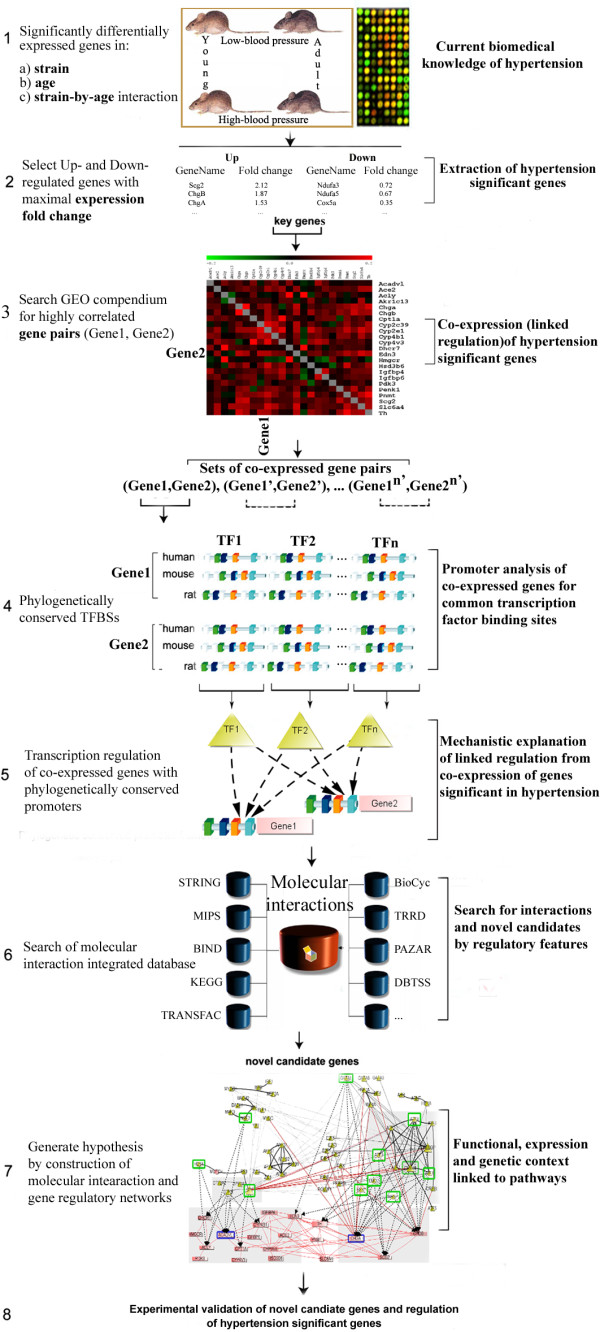
**Systems analysis in BiologicalNetworks.**The figure details the strategy that was used to systematically search for co-expressed (co-regulated) genes and promoter frameworks linked to the co-regulation of genes significant in hypertension in human, mouse and rat.

In this section, we demonstrate a case study of the search for potential therapeutic targets for hypertension. Specifically, it is shown how, using BiologicalNetworks and starting with a single microarray experiment in the model organism *Rattus norvegicus *[39], one can identify regulatory regions in the hypertension essential genes and investigate transcriptional modules and gene regulatory networks describing multi-factorial nature of hypertension (Figure 6). Below the case study is described step-by-step.

First, among about 1000 genes significantly perturbed in hypertension in the microarray experiment [39] (Figure 6-1), we found 25 over-expressed and 110 under-expressed genes that showed the maximal fold change of expression in hypertension (Table S1 in Additional File 1) (Figure 6-2). For the demonstration purpose of this study, we focused only on over-expressed genes. Further, using the multi-experiment/multi-gene Microarray Search Panel, we searched the microarray experiments for co-expressed pairs containing the selected over-expressed genes (Figure 6-3). Data in every experiment was normalized; that is, the expression vector was subtracted with the mean of the expression values in the experiment and divided by the standard deviation. Since different experiments have different number of time points and conditions, calculation of Pearson correlation coefficient (PCC) was FDR-corrected, so that calculated p-values for PCC took into account the length of the expression vectors (experiments). For further analysis, only pairs that were co-expressed in more than 10 experiments were chosen (Figure S3, Section S1.4, in Additional File S1).

**Figure 7:**
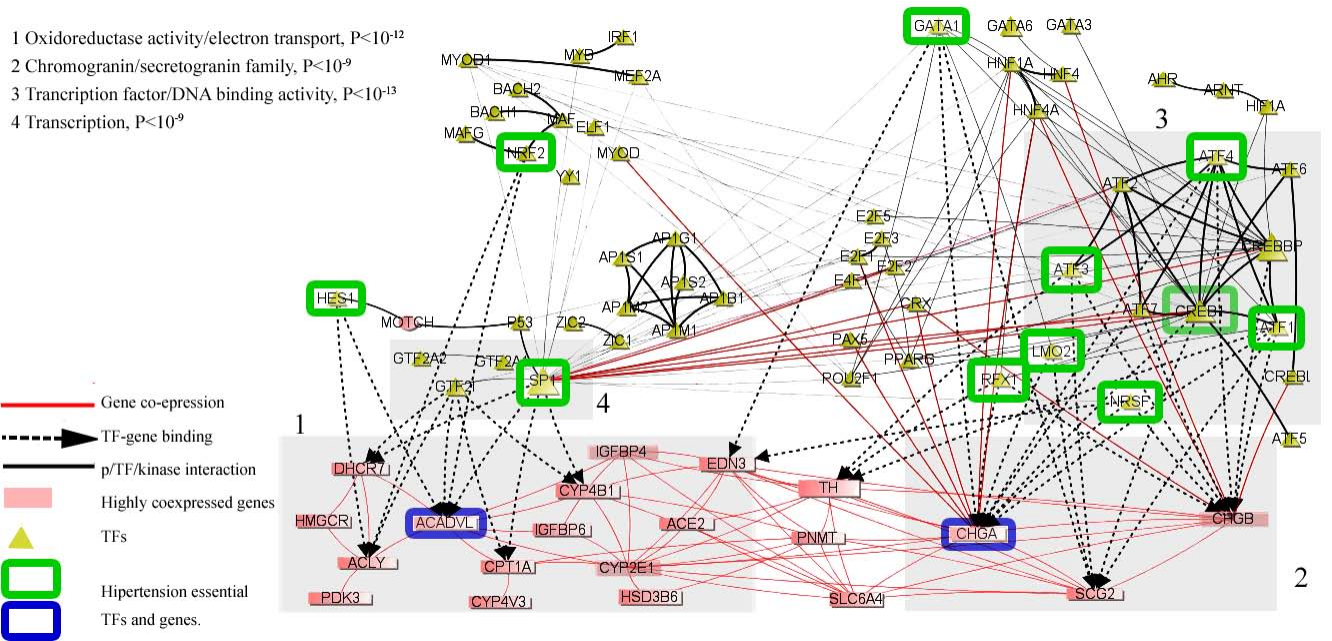
**Integrated molecular interaction network of human/mouse/rat hypertension**. **Red rectangles **- highly co-expressed (red lines) and over-expressed genes in hypertension. **Yellow triangles **- TFs potentially regulating hypertension-specific genes, which TF binding sites are conserved in human, mouse and rat. **Black lines **- physical protein/TF/kinase - protein/TF/kinase interactions. **Green and Blue squares **- genes/proteins/TFs that are well known from literature to be essential in hypertension. **Red lines **- co-expressed genes/proteins. **Dashed lines **- predicted TFs and their target genes. Numbers (1-4) correspond to gene/protein groups functionally enriched by particular GO terms, shown in the upper left corner.

Second, we built a network of 20 selected genes, selected at the previous step, that were co-expressed and at the same time over-expressed in hypertension, together with TFs that might potentially regulate transcription of these genes [40]. To search for TFs, we considered that gene regulation is controlled to a significant degree by TFBSs within proximal promoters and the fact that in orthologous promoters, the relative order and spacing among TFBSs expected to be conserved during evolution [22-24]. For the 20 selected genes, we first identified orthologous promoter regions in human, mouse and rat (Figure 6-4). We considered the regions from -6 kb to 500 bp relative to the transcription start sites and used Comparative Genomics Browser, which was described in the above section and shown in Figure 4. Then, we searched for TFBSs that were conserved in the orthologous promoters (Figure 6-5). Binding sites were filtered at p-values below 10^-3 ^and examined visually for consistency. In the result, we obtained 103 TFs that might potentially regulate transcription of 18 hypertension-specific genes (Figure S3B, 3C, Section S1.5 in Additional File S1). To construct the final network, we searched for all reported interactions among identified 103 TFs and 20 genes (Figure 6-6). The obtained network is shown in Figure 7; it consists of 78 TFs/proteins/genes since depicts only those of analyzed TFs/proteins/genes for which at least one pairwise interaction/relation was reported in the databases integrated in Biological-Networks.

Third, to investigate the constructed network, we searched the GEO compendium for co-expressed TF-gene pairs; we found pairs that are connected by red lines in Figure 7. We also investigated whether more disease information related to the found genes/TFs can be obtained from OMIM database. Using the keyword search mechanism, we first searched the integrated database for MeSH terms related to 'hypertension' and then searched for these terms in the OMIM database. Among all human genes, hypertension-associated MeSH terms were found in 504 genes, or about 1.6% of all human genes. While in the set of 103 transcription factor found in this study (20 genes were not considered), 32 genes, or 31%, had hypertension associated terms (Table S2 in Additional File S1).

Figure 7 shows the integrated picture, visualizing in BiologicalNetworks, of the network of found genes/proteins/TFs and interactions among them. Well-known genes and TFs associated with hypertension are depicted in blue and green squares in Figure 7; while other genes/TFs can be suggested for further experimental investigation on association with hypertension and considered as drug target candidates for hypertension. Similar analysis can be done, using the genes that were under-expressed in the considered microarray experiment. The described project can be seen and further analyzed launching BiologicalNetworks and opening 'BMC Bioinformatics Demo Project'.

## Conclusions

The new release of BiologicalNetworks introduces extensive functionality for a more efficient integrated analysis and visualization of diverse data in studies of different biological systems concerning human diseases, host-pathogen interactions, metagenomics, meiosis in fungi, microbial metabolism, and whole-genome metabolic reconstruction in eukaryotes and prokaryotes. The BiologicalNetworks database has a general purpose graph architecture and is data-type neutral, thus there is the prospect of further data integration for more complete systems biology studies. The integration of additional, orthogonal sources of information, such as clinical data, will enable quantitative associations of clinical variables with the activities of molecular pathways and processes. We also demonstrated how BiologicalNetworks can be used to find disease-specific interaction networks, through the application of multi-level analysis of microarray, sequence, regulatory, and other data.

Besides customization on the level of selecting analysis methods/tools in BiologicalNetworks, the user has an option to change the parameters of each method; for example, specify the homology level in the "Build Homology Wizard" when building the clusters of homologous genes/proteins or specify data sources, types of interactions, species, and p-values in the "Build Pathway Wizard". We are also customizing BiologicalNetworks constantly adding new features, methods, data formats and sources by the users' requests.

To allow for the replication and comparison of the results presented in this work with other related analysis, all available demonstrated examples and data can be accessed in 'BMC Bioinformatics Demo Project', upon launching the BiologicalNetworks application. Additionally, the BiologicalNetworks Welcome Screen and front page of the web site contains a list of "driving" biological projects (for various species and types of analysis) which can be replicated by simply running the respective project.

BiologicalNetworks, along with the user Manual and Video tutorials and Quick Start Guide, is available at http://www.biologicalnetworks.org.

## Availability and requirements

**Project name**: BiologicalNetworks

**Project home page**: http://www.biologicalnetworks.org

**Operating systems**: Windows 2000/XP/Vista/7, Linux/Ubuntu/Redhat, MacOSX

**Programming language**: Java

**License**: Free for academic purposes

**Other requirements**: 2GB RAM

## Authors' contributions

MB, AG and MS contributed to system concept. SK, YD, MS and MB implemented the system and performed major programming work. MB and JP coordinated this work, contributed to data analysis and wrote the manuscript. All authors read and approved the final manuscript.

## Supplementary Material

Additional file 1 **Methods**. Detailed description of the methods and data types used in the BiologicalNetworks system. Click here for file
