# Supplementary material for: BiologicalNetworks 2.0 - an integrative view of genome biology data
Source: BMC Bioinformatics. 2010 Dec 29;11:610. doi: 10.1186/1471-2105-11-610 (PMC3019228; doi:10.1186/1471-2105-11-610)
Supplement: Additional file 1 — Methods. Detailed description of the methods and data types used in the BiologicalNetworks system. [file 1471-2105-11-610-S1.DOC]

## Supplementary File S1

**S1.1 BioNets Ontology**

BioNets ontology (Fig. S1) consists of the general-purpose Basic Ontology that was manually developed and that maps the classes from different domains; for example, protein, gene, pathway, interaction, disease, cell, tissue, drug, chromosome, COG functional group, gene set (e.g., operon, regulon). Currently, Basic Ontology is manually mapped onto 25 OBO ontologies, including Sequence Ontology, GeneOntology, Human Disease, CheBI, BRENDA Tissues. These 25 ontologies were selected as the ontologies that are curated and regularly updated. Thanks to the efforts of OBO consortium ([www.bioontology.org](http://www.bioontology.org/)) that provides the mapping among more than 200 ontologies, we were able to automatically integrate in the BioNets ontology 98 ontologies total – as new databases will be integrated in our system more ontologies will be added, if needed. The basic.owl file with Basic Ontology and mappings from it to other ontologies can be downloaded at <http://www.integromedb.org/bionetsonto.php>.


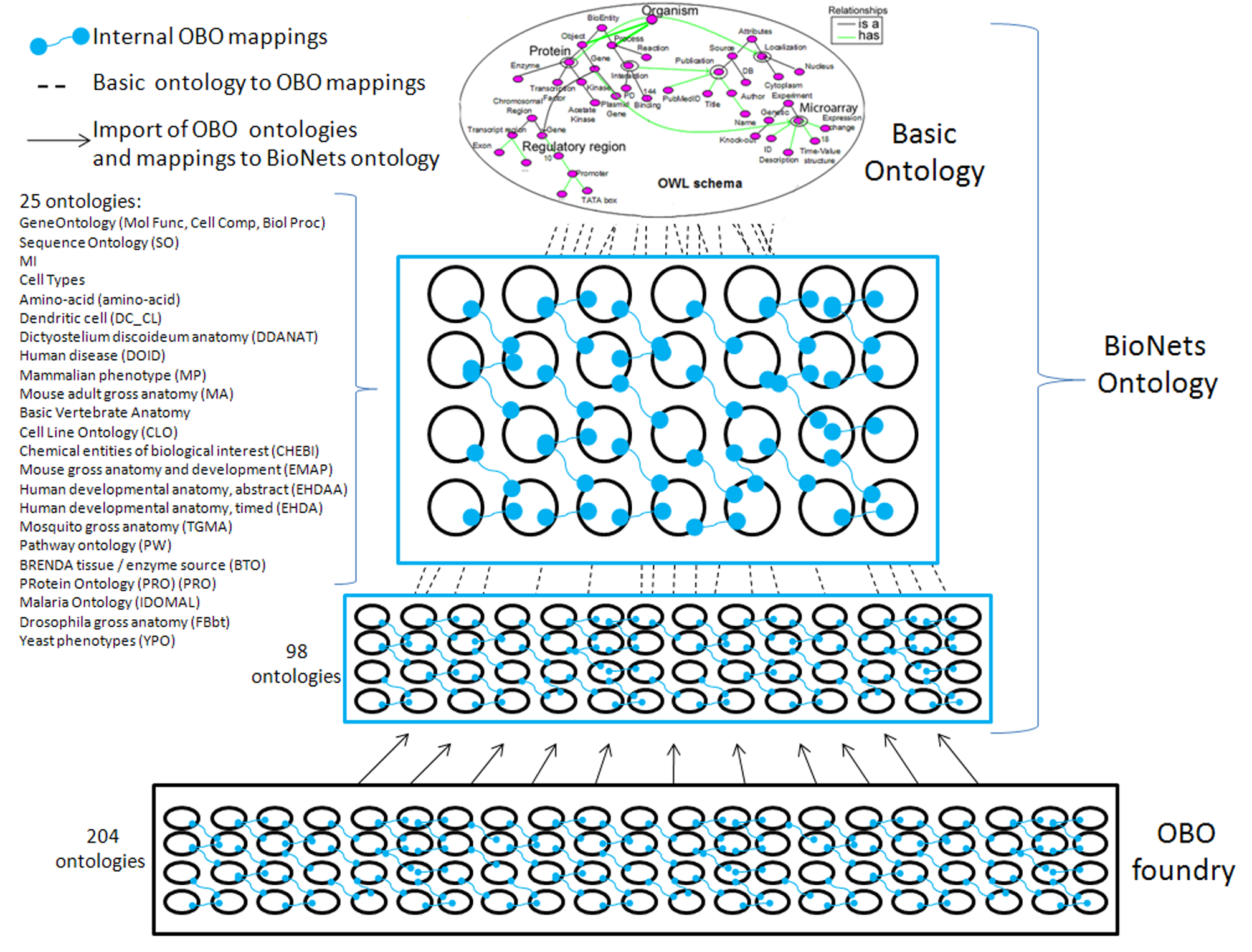


**S1. Schema of integration of OBO ontologies into BioNets ontology.**

**S1.2 User’s Data Integration**

Any public or user’s data in the tab-delimited format can be integrated into BiologicalNetworks (Fig. S2) database and studied together with other already integrated data.

S2. User’s Data Integration page allows integrate host-pathogen data which is interesting for particular user, but we didn’t integrated. The integration procedure consists of three easy steps: 1) User registration, 2) Mapping of the data and 3) Integration of the data.


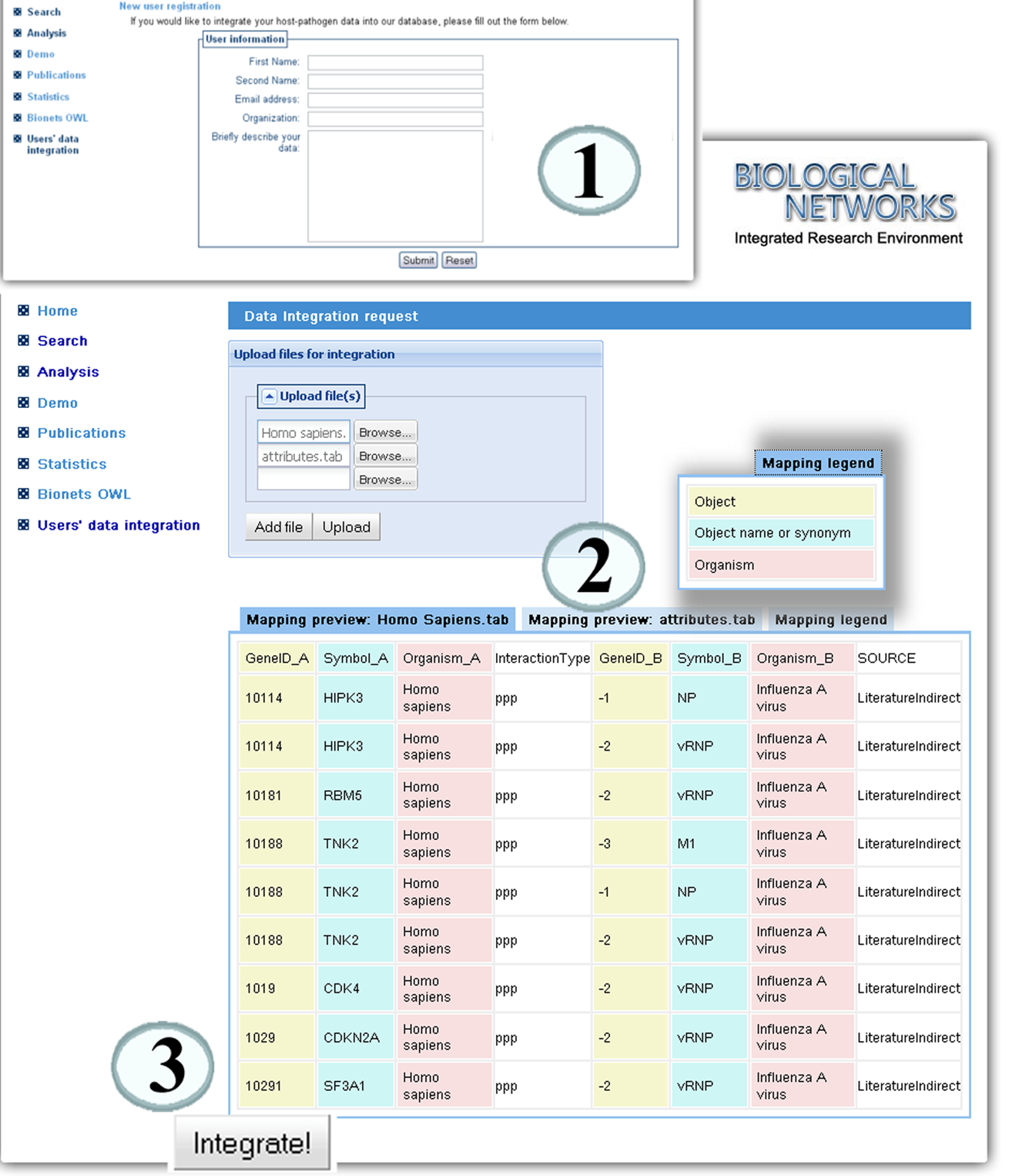


**S1.3 Demonstration Study**

Gene regulation program of the cell is controlled to a significant degree by functional elements within their proximal promoter regions represented by the nucleotide sequence immediately upstream from the site of transcriptional initiation, often overlapping with the respective transcribed sequence. It has been proposed that convergent evolution leading to organizational similarities of regulatory elements among different promoters provides a potential mechanism for the synchronization of the expression of genes whose products must interact within a common biological process (5, 6) and orchestrates in time and space regulatory mechanisms required for complex structures and processes (8, 9). The relative order and spacing of regulatory elements in promoter modules (promoter framework) (5, 7), such as transcription factor (TF) binding sites (TFBSs), are often highly conserved through evolution, highlighting their importance in regulation. Recent methods offer rapid identification of these sets of elements through comparative genomics, an approach similar to phylogenetic footprinting (10).

| **#** | **Name** | **Descripion** |
| --- | --- | --- |
| 1 | Acadvl | acyl-Coenzyme A dehydrogenase, long chain |
| 2  4 | Ace2 | angiotensin I converting enzyme |
| 3 | Acly | ATP citrate lyase |
| 4 | Akr1c13 | aldo-keto reductase family 1, member C13 |
| 5 | Chga | chromogranin A |
| 6 | Chgb | chromogranin B |
| 7 | Cpt1a | carnitine palmitoyltransferase 1a, liver |
| 8 | Cyp2c39 | cytochrome P450, family 2, subfamily c |
| 9 | Cyp2e1 | cytochrome P450, family 2, subfamily e |
| 10 | Cyp4b1 | cytochrome P450, family 4, subfamily b |
| 11 | Cyp4v3 | cytochrome P450, family 4, subfamily v |
| 12 | Dhcr7 | 7-dehydrocholesterol reductase |
| 13 | Edn3 | endothelin 3 |
| 14 | Facl4 | acyl-CoA synthetase long-chain family member 4 |
| 15 | Gch | GTP cyclohydrolase 1 |
| 16 | Hmgcr | 3-hydroxy-3-methylglutaryl-Coenzyme A reductase |
| 17 | Hsd3b6 | hydroxysteroid dehydrogenase-6 |
| 18 | Igfbp4 | insulin-like growth factor binding protein 4 |
| 19 | Igfbp6 | insulin-like growth factor binding protein 6 |
| 20 | Pdk3 | pyruvate dehydrogenase kinase, isoenzyme 3 |
| 21 | Penk1 | preproenkephalin 1 |
| 22 | Pnmt | phenylethanolamine-N-methyltransferase |
| 23 | Scg2 | secretogranin II |
| 24 | Slc6a4 | solute carrier family (neurotransmitter transporter, serotonin) |
| 25 | Th | tyrosine hydroxylase |

**Table S1.** Top overexpressed mouse genes, differentially expressed in Hypertension, included in analysis.

To test these hypotheses, a unique tissue environment linked to a genetic disease, such as hypertension, was selected for a systems biology -driven analysis as outlined in [Fig.](http://www.pnas.org/content/103/15/5682.full#F1) 6. The genetic basis of hypertension in the genetically/hereditary hypertensive (BPH/2) strains is incompletely understood (11, 12) and to our knowledge presently no one study reports about a general approach to identify regulatory networks of hypertension describing multifactorial nature of this condition.

Adrenal gland secretory products, both medullary and cortical, are logical candidates for study in hypertension because they directly influence endocrine, cardiovascular, and sympathetic function. Epinephrine and norepinephrine act through G-protein– coupled adrenergic receptors to affect sympathetic functions, such as the force of contraction of the heart and constriction of blood vessels. Adrenal cortical mineralocorticoid hormones regulate the reabsorption and secretion of sodium and potassium and can therefore also modulate blood pressure. Study of the adrenal neuroendocrine transcriptome, therefore, might be an efficient way to investigate many candidate genes for hypertension simultaneously. The purpose of this study is to use global gene expression patterns in the adrenal gland and analyze the transcriptional regulatory sequences in the proximal promoters in mammalian species to explore the genetic basis of hypertension in an attempt to gain insight into gene regulatory and molecular mechanisms that govern human essential hypertension.

### S1.4 Identification of functionally related or co-regulated pairs of genes significant in hypertension.

Genes exhibiting correlated expression patterns may be co-regulated by common transcription factors or be functionally related, forming a functional module or a molecular complex. In an attempt to address this hypothesis for genes significant in hypertension, a systematic large scale search over GEO experiments was applied to identify pairs of genes that are co-expressed in different biological systems and are differentially expressed in hypertension.


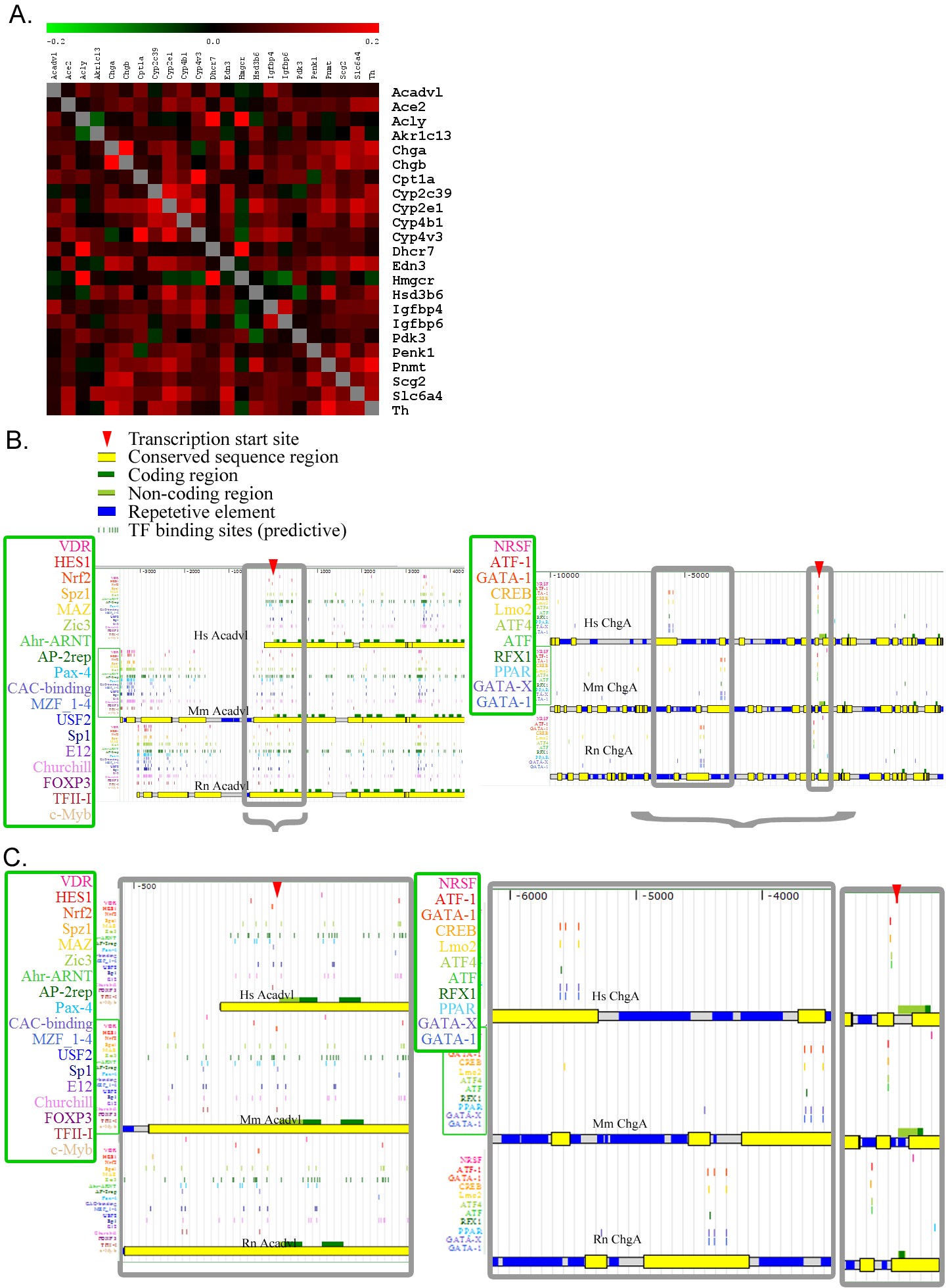


**Fig S3.**

**A)** Pearson correlation expression distance matrix among 25 top overexpressed genes in Hypertension, built on GEO compendium search

**B)** Evolutionary conserved predicted transcription factor binding sites for genes essential for hypertension. 15-kb proximal promoter region is displayed, exons, introns, the transcription start site, repetitive elements, conserved sequence regions and transcription factor binding sites, are color-coded. Transcription factor binding sites for Acadvl and ChgA genes conserved in Human, Mouse and Rat.

**C)** Detailed view of transcription factor binding sites on Acadvl and ChgA genes.

Differentially expressed genes showing strain and age differences, and strain-by-age interaction were identified in previous studies of hypertension (11, 12). As described in Materials and Methods, 25 overexpressed and 110 underexpressed genes which have the maximal expression fold change in hypertension were first included for analysis (Table 1). For chosen gene lists we mined publicly available GEO compendium of mouse and human microarray data (gene expression over many different conditions and time points) for gene pairs that are co-expressed in different biological systems and are significantly perturbed in Hypertension. Data in every experiment is normalized, so every expression vector is subtracted with mean and divided by standard deviation of the expression values in a experiment. Since different experiments have different number of time points and conditions Pearson correlation calculation was FDR corrected, so that calculated p-Values for PCC take into account the length of the expression vectors (experiments). The correspondent Pearson correlation similarity matrix for gene pairs and respective pValues are represented on Fig S2A. Once we obtain pValues for every PCC, we rank them from smallest to largest (P1, P2, …, Pm), we chose a significance level as s=0.01, found largest k such that Pk<=k*s/m. Thus we obtained k=151 values as significant (16) corresponding to FDR corrected P <4*10-3. Large scale Pearson correlation calculation ([Fig. 1](http://www.pnas.org/content/103/15/5682.full#F1), steps 1–3). of gene pairs over ~600 GEO experiments were performed using PathSys data integration system and BiologicalNetworks analysis environment.

### S1.5 Identification of Evolutionarily Shared Promoter Structures in hypertension significant Genes.

In orthologous promoters, elements important for regulation of a given gene are expected to be conserved over evolution. Starting with a set of gene pairs found to be differentially expressed in hypertension and significantly co-regulated or functionally related by GEO compendium search, orthologous promoter regions were identified from three species (*Homo sapiens, Mus musculus,* and *Rattus norvegicus*) by using a comparative genomics tool. Out of 25 significantly co-expressed genes associated with hypertension, for 18 promoter sequences from at least two species could be identified.

Genes encoding proteins that functionally interact may exhibit conserved organization of promoter elements ([5](http://www.pnas.org/content/103/15/5682.full#ref-5)). In an attempt to address this hypothesis for hypertension-associated genes, a systematic approach was applied to identify promoter frameworks shared among hypertension - overexpressed genes. As described in Materials and Methods, TFBSs common to promoter regions across species were first identified for individual genes ([Table 1](http://www.pnas.org/content/103/15/5682.full#T1)). Using modification of phylogenetic footprinting method, for our set of co-expressed genes, we searched for transcription factor binding sites, that are enriched in the region of 6000 bases upstream of each transcription start site to 500 bases downstream of each translation start of every gene in the gene pairs found and are conserved in Mouse, Rat and Human genomes. Binding sites and correspondent TFs were filtered for P<10-3 and visually examined for consistency (Fig. S2B, C).

Parameter settings of TFBS selection, strand orientation, and order were strictly determined by sequence analysis of the promoter sets. There were only two parameters that were subsequently optimized manually. Stringency of evolutionary conservation by selecting a subset of the six available species, which plays a central role in analysis because only transcription factor binding sites that are conserved in the selected species are scored in the analysis. Minimal distance ranges between matrices were reduced (from default 10 nt to 1 nt) to include conserved TFBSs identified by visual inspection. Matrix similarities were used at default values and fractionally adjusted (default −0.1, −0.2, or −0.5) when the reduction allowed detection of evolutionary conserved TFBS sets that were missed with the default settings. The evolutionary conserved frameworks were determined by using modification of phylogenetic footprinting method on the basis of the predefined TFBS subsets. Models, reflecting the frameworks, were then built by using PathSys data integration system and BiologicalNetworks analysis environment and optimized for all three species, ([Fig.](http://www.pnas.org/content/103/15/5682.full#F1) 6, steps 1–5).

**Fig S4.** Integrated molecular interaction network of mouse Hypertension. Red rectangles- highly coexpressed (red lines) overexpressed genes in Hypertnsion. 2) Yellow triangles - TFs having binding sites (conserved in Hs, Rn, Mm). 3) Black lines - physical (p/TF/Kinase - p/TF/kinase) interaction. 4) Green squares- examples of TF that have binding sites represented of fig a). 5) Blue squares- examples of target genes having conserved binding sites and represented on fig b). 6) Red lines - coexpressed genes/proteins. 7) Dashed lines- predicted binding of TF and target genes.


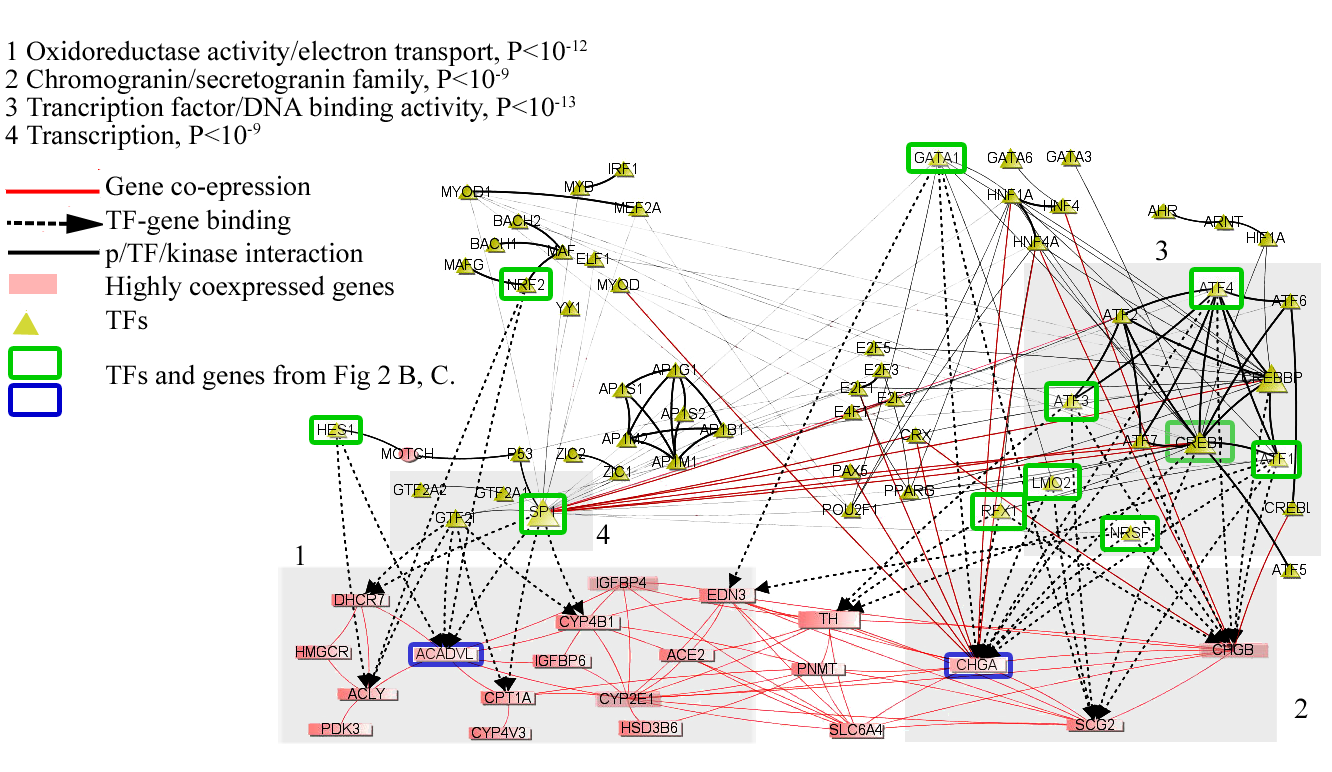


This approach eventually yielded 103 potential transcription factors were found for our set of 18 genes and promoter frameworks of conserved TFBSs that originated in hypertension specific genes and was also found in the human, mouse, and rat promoters ([Fig.](http://www.pnas.org/content/103/15/5682.full#F1) 6, step 5) by searching the hypertension-associated proximal promoter sequences (total of 47 sequences, see [Table 1](http://www.pnas.org/content/103/15/5682.full#T1)).

For discovering novel potential interactors of hypertension significant genes and transcription factors we used BiologicalNetworks integrated database (14, 15) dedicated to molecular interactions, including both physical and functional interactions among genes and proteins. It weights and integrates information from numerous sources, including experimental repositories, computational prediction methods and public text collections, thus acting as a meta-database that maps all interaction evidence onto a common set of genomes and proteins.

We searched the integrated database for protein-protein physical interaction, phosphorulation events all existing protein-protein (TF-TF, TF-kinase, p-p) interactions.

For newly found transcription factors and proteins we searched the GEO compendium to find which of these potential interactors are co-expressed with any of transcription factors or are co-regulated with target genes. The integrated picture of co-expressed genes and proteins together with their interactors and bindings of transcription factors to their target genes is represented on Figure 3.

|  | **TF names** | **Description** | **Associated disease** |
| --- | --- | --- | --- |
| 1 | NPPA, PND, ANP, ATFB6 | Natriuretic peptide precursor A | "Atrial fibrillation, familial, 6, 612201 (3)" |
| 2 | PAX8 | Paired box homeotic gene-8 | "Hypothyroidism, congenital, due to thyroid dysgenesis or" |
| 3 | SP110, IFI41, IFI75, VODI | "SP110 nuclear body protein (interferon-induced protein 41, 30kD;" | "Hepatic venoocclusive disease with immunodeficiency, 235550 (3);" |
| 4 | PPARG, PPARG1, PPARG2 | "Peroxisome proliferator activated receptor, gamma" | "Obesity, severe, 601665 (3); [Obesity, resistance to] (3);" |
| 5 | CASR, HHC1, PCAR1, FIH | Calcium-sensing receptor | "Hypocalciuric hypercalcemia, type I, 145980 (3); Hyperparathyroidism," |
| 6 | PPARGC1A, PPARGC1 | "Peroxisome proliferator-activated receptor-gamma, coactivator 1, alpha" | "Lipodystrophy, familial partial, with decreased subcutaneous fat" |
| 7 | ATFB5 | "Atrial fibrillation, familial, 5" | "{Atrial fibrillation, familial, 5} (2)" |
| 8 | ELMOD2 | ELMO domain-containing protein 2 | "{Pulmonary fibrosis, idiopathic}, 178500 (2)" |
| 9 | HAND2, DHAND2, DHAND | Heart- and neural crest derivative-expressed 2 | NULL |
| 10 | CART | Cocaine- and amphetamine-regulated transcript | "{Obesity, susceptibility to}, 601665 (3)" |
| 11 | PPARGC1B, PGC1B, PERC | "Peroxisome proliferator-activated receptor-gamma, coactivator 1, beta" | "{Obesity, variation in}, 601665 (3)" |
| 12 | NKX2E, CSX | "NK2 transcription factor, Drosophila, homolog of, E" | "Atrial septal defect with atrioventricular conduction defects," |
| 13 | GCMB | "Glial cells missing, Drosophila, homolog of, B" | "Hypoparathyroidism, familial isolated, 146200 (3)" |
| 14 | KCNK16, TALK1 | "Potassium channel, subfamily K, member 16" | NULL |
| 15 | KCNK17, TASK4, TALK2 | "Potassium channel, subfamily K, member 17" | NULL |
| 16 | FCYT, PKHD1, ARPKD | Fibrocystin | "Polycystic kidney and hepatic disease, 263200 (3)" |
| 17 | ATFB2 | "Atrial fibrillation, familial, 2" | "Atrial fibrillation, familial, 2 (2)" |
| 18 | GLIS3, ZNF515 | GLIS family zinc finger protein 3 | "Diabetes mellitus, neonatal, with congenital hypothyroidism, 610199" |
| 19 | FOXE1, FKHL15, TITF2, TTF2 | Forkhead box E1 (thyroid transcription factor-2) | "Bamforth-Lazarus syndrome, 241850 (3)" |
| 20 | GATA3, HDR | GATA-binding protein-3 | "Hypoparathyroidism, sensorineural deafness, and renal dysplasia," |
| 21 | ATFB1 | "Atrial fibrillation, familial, 1" | "Atrial fibrillation, familial, 1 (2)" |
| 22 | CSPG6, SMC3, HCAP, BAM, CDLS3 | Chondroitin sulfate proteoglycan 6 | "Cornelia de Lange syndrome 3, 610759 (3)" |
| 23 | KCNQ1, KCNA9, LQT1, KVLQT1, ATFB1, SQT2 | "Potassium voltage-gated channel, KQT-like subfamily, member 1" | "Long QT syndrome-1, 192500 (3); Jervell and Lange-Nielsen syndrome," |
| 24 | MYBPC3, CMH4 | "Myosin-binding protein C, cardiac" | "Cardiomyopathy, familial hypertrophic, 4, 115197 (3);" |
| 25 | KCNA5, ATFB7 | "Potassium voltage-gated channel, shaker-related subfamily, member 5" | "Atrial fibrillation, familial, 7, 612240 (3)" |
| 26 | THRAP2, PROSIT240, TRAP240L, KIAA1025 | Thyroid hormone receptor-associated protein 2 | "Transposition of the great arteries, dextro-looped 1, 608808 (3)" |
| 27 | MEF2A, ADCAD1 | "MADS box transcription enhancer factor 2, polypeptide A (myocyte" | "Coronary artery disease, autosomal dominant, 1, 608320 (3)" |
| 28 | PRKAR1A, TSE1, CNC1, CAR, PPNAD1 | "Protein kinase, cAMP-dependent, regulatory, type I, alpha" | "Carney complex, type 1, 160980 (3); Myxoma, intracardiac, 255960 (3);" |
| 29 | SLC9A3R1, EBP50, NHERF | "Solute carrier family 9 (sodium/hydrogen exchanger), isoform 3" | NULL |
| 30 | TRAP240 | "Thyroid hormone receptor-associated protein, 240kD subunit" | NULL |
| 31 | SCN1B, GEFSP1 | "Sodium channel, voltage-gated, type I, beta polypeptide" | "Generalized epilepsy with febrile seizures plus, 604233 (3)" |
| 32 | ARSE, CDPX1, CDPXR | Arylsulfatase E | "Chondrodysplasia punctata, X-linked recessive, 302950 (3)" |
| **Table S2.** Transcription factors predicted to be significant for hypertension that have  ‘hypertension’ associated OMIM terms. | | | |

### S1.6 Identification of disease associated terms in OMIM and databases.

We investigated whether we could obtain more disease information related to the focused genes by searching OMIM (Online Mendelian Inheritance in Man) database by inputting disease MeSH terms. Table S2 lists OMIM hypertension related terms. We searched OMIM database for gene-disease associations among transcription factors found to be significant for hypertension and MeSH terms. Out of 11304 total OMIM genes hypertension associated terms are found in 504 which is 4,9% percent of all OMIM genes; in our set of 103 transcription factor 32 (31%) genes have hypertension associated terms, which is a significant enrichment.

**REFERENCES**

1. Tenen DG. Disruption of differentiation in human cancer: AML shows the way. *Nat. Rev. Cancer*, ( (2003) ) **3**, : 89–101.
2. Mooradian AD, Haas MJ, Wong NC. Transcriptional control of apolipoprotein A-I gene expression in diabetes. *Diabetes*, ( (2004) ) **53**, : 513–520.
3. Davicioni E, Finckenstein FG, Shahbazian V, Buckley JD, Triche TJ, Anderson MJ. Identification of a PAX-FKHR gene expression signature that defines molecular classes and determines the prognosis of alveolar rhabdomyosarcomas. *Cancer Res*, ( (2006) ) **66**, : 6936–6946.
4. Borovecki F, Lovrecic L, Zhou J, Jeong H, Then F, Rosas HD, Hersch SM, Hogarth P, Bouzou B, et al. Genome-wide expression profiling of human blood reveals biomarkers for Huntington's disease. *Proc. Natl Acad. Sci.* USA, ( (2005) ) 102, : 11023–11028.
5. Fessele, S., Maier, H., Zischek, C., Nelson, P. J. & Werner, T. (2002) Trends60–63.
6. Liu, R., McEachin, R. C. & States, D. J. (2003) Genome Res. 13, 654–661.
7. Dohr, S., Klingenhoff, A., Maier, H., Hrabe de Angelis, M., Werner, T. & Schneider,(2005) Nucleic Acids Res. 33, 864–872.
8. Qiu, P., Qin, L., Sorrentino, R. P., Greene, J. R., Wang, L. & Partridge, N. C. (2003) J. Mol. Biol. 326, 1327–1336.
9. Halfon, M. S., Grad, Y., Church, G.M. &Michelson, A. M. (2002) Genome Res. 12, 1019–1028.
10. Qiu, P., Qin, L., Sorrentino, R. P., Greene, J. R., Wang, L. & Partridge, N. C. (2003) J. Mol. Biol. 326, 1327–1336.
11. Fries RS, Mahboubi P, Mahapatra NR, Mahata SK, Schork NJ, Schmid-Schoenbein GW, O’Connor DT. 2004. Neuroendocrine transcriptome in genetic hypertension: multiple changes in diverse adrenal physiological systems. Hypertension 43, 1301-1311.
12. Friese RS, Mahboubi P, Mahapatra NR, Mahata SK, Schork NJ, Schmid-Schoenbein GW, O’Connor DT. 2005. Common genetic mechanisms of blood pressure elevation in two independent rodent models of human essential hypertension. Am J Hypertens  18, 633-652.
13. Baitaluk, M., Qian, X., Godbole, S., Raval, A., Ray, A. and Gupta, A. PathSys: Integrating molecular interaction graphs for systems biology. BMC Bioinformatics 7(55) doi: 10.1186/1471-2105-7-55. (2006).
14. Baitaluk, M., Sedova, M., Ray, A. and Gupta, A. Biological networks: Visualization and analysis tool for systems biology. Nucleic Acid Res. 34 (Web Server Issue), W466-71, 2006.
15. 16. Benjamini, Yoav; Hochberg, Yosef (1995). "[Controlling the false discovery rate: a practical and powerful approach to multiple testing](http://www.math.tau.ac.il/~ybenja/MyPapers/benjamini_hochberg1995.pdf)". [Journal of the Royal Statistical Society](http://en.wikipedia.org/wiki/Journal_of_the_Royal_Statistical_Society), Series B (Methodological) 57 (1): 289–300.
